# Supplementary figures and images for: Glycine cleavage system determines the fate of pluripotent stem cells via the regulation of senescence and epigenetic modifications
Source: Life Sci Alliance. 2019 Sep 27;2(5):e201900413. doi: 10.26508/lsa.201900413 (PMC6765226; doi:10.26508/lsa.201900413)

**Fig 1F**

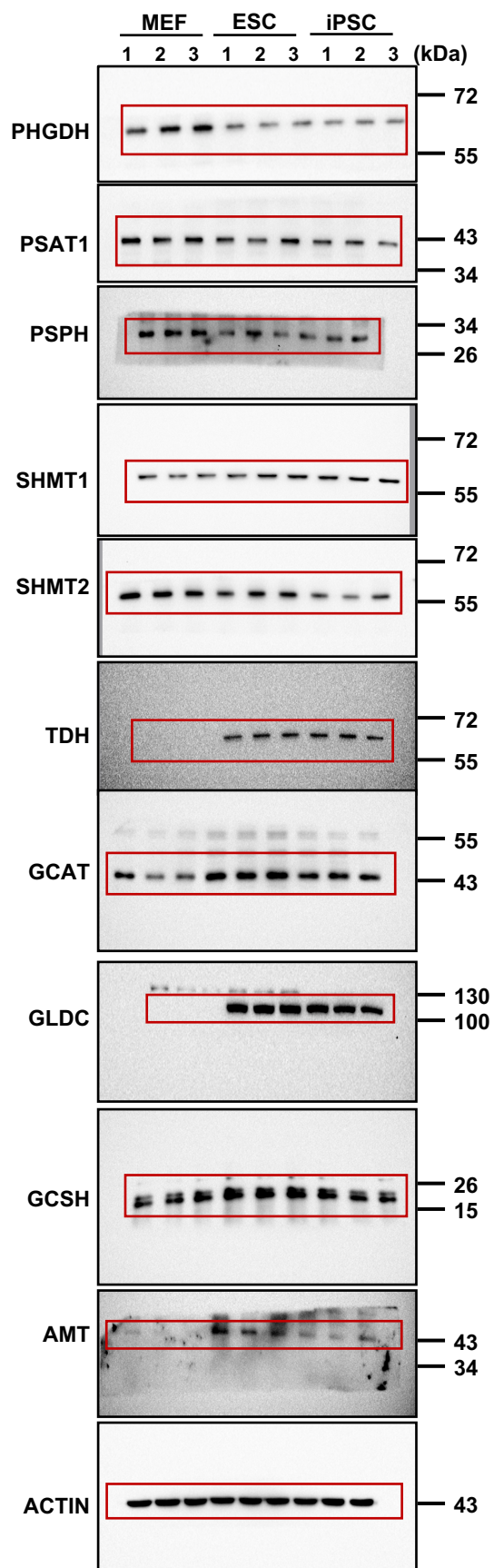

Supplement: Supplementary file 1 [file LSA-2019-00413_SdataF1.pdf]

**Fig S1B**

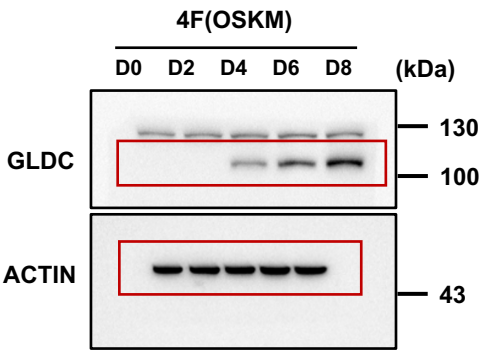

**Fig S1C**

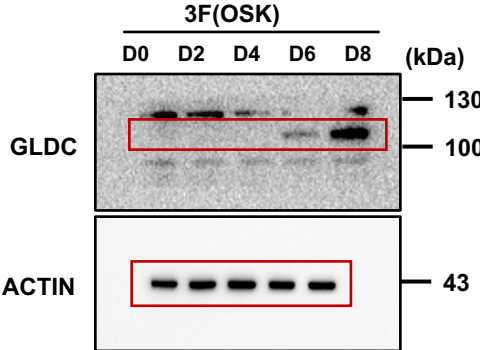

**Fig S1D**

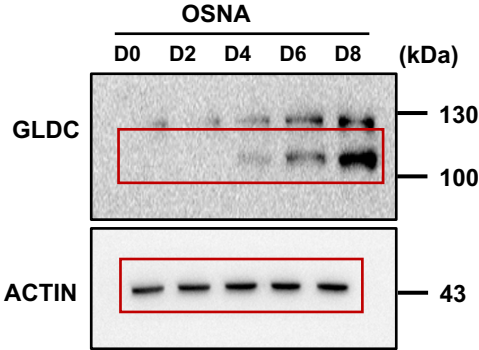

**Fig S1E**

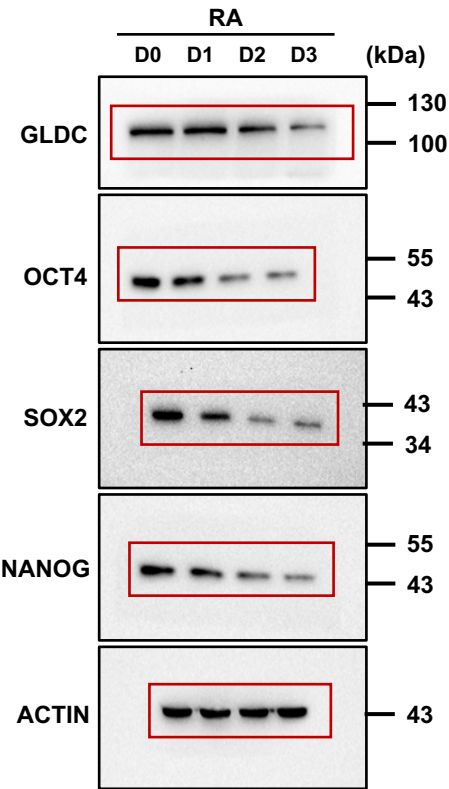

Supplement: Supplementary file 2 [file LSA-2019-00413_SdataFS1.pdf]

# Fig 2A

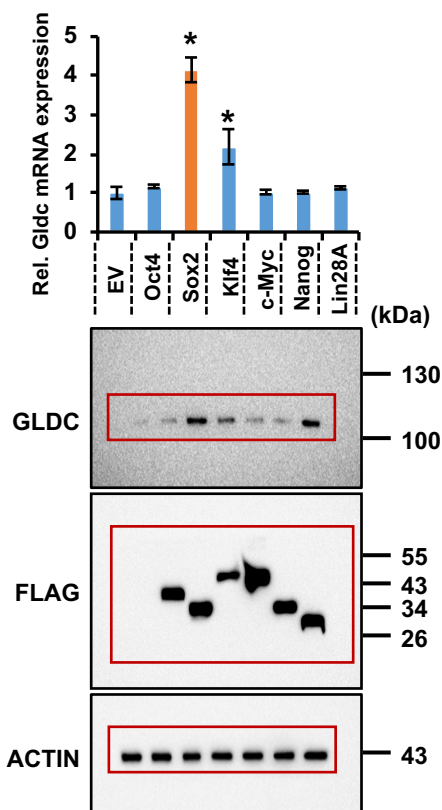

# Fig 2B

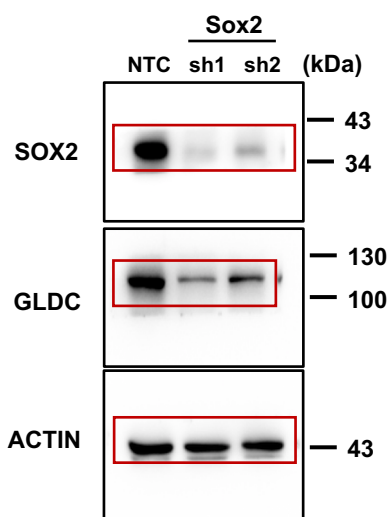

# Fig 2F

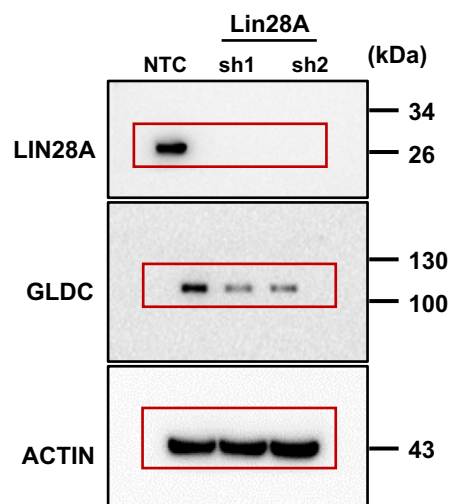

# Fig 2H

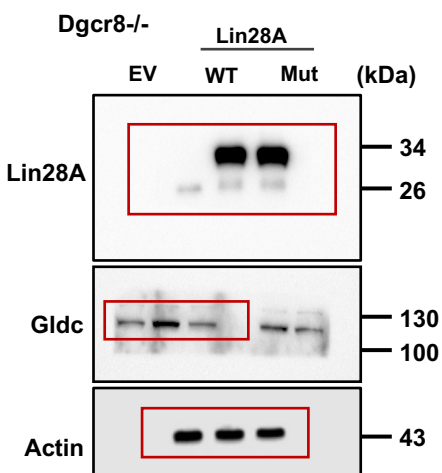

# Fig 2M

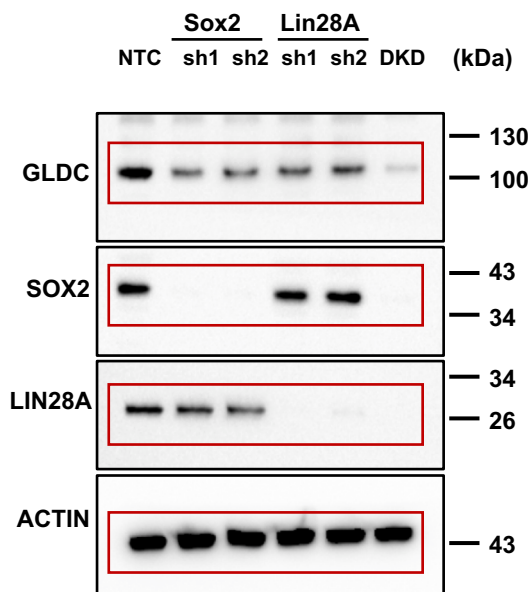

Supplement: Supplementary file 3 [file LSA-2019-00413_SdataF2.pdf]

**Fig S2C**

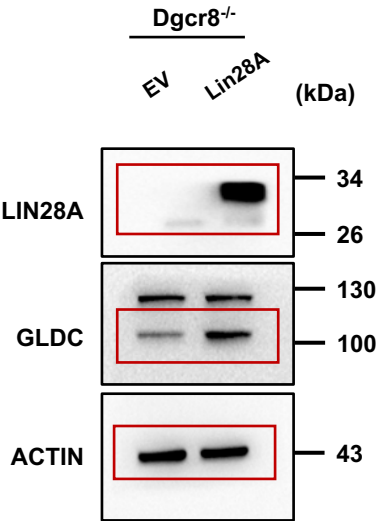

Supplement: Supplementary file 4 [file LSA-2019-00413_SdataFS2.pdf]

**Fig S3A**

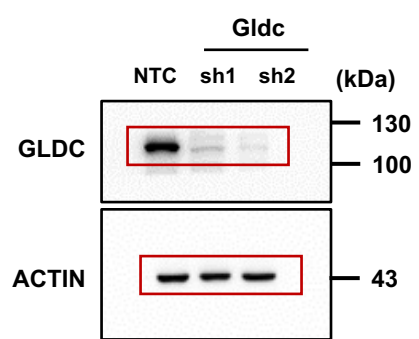

Supplement: Supplementary file 5 [file LSA-2019-00413_SdataFS3.pdf]

Fig 4C

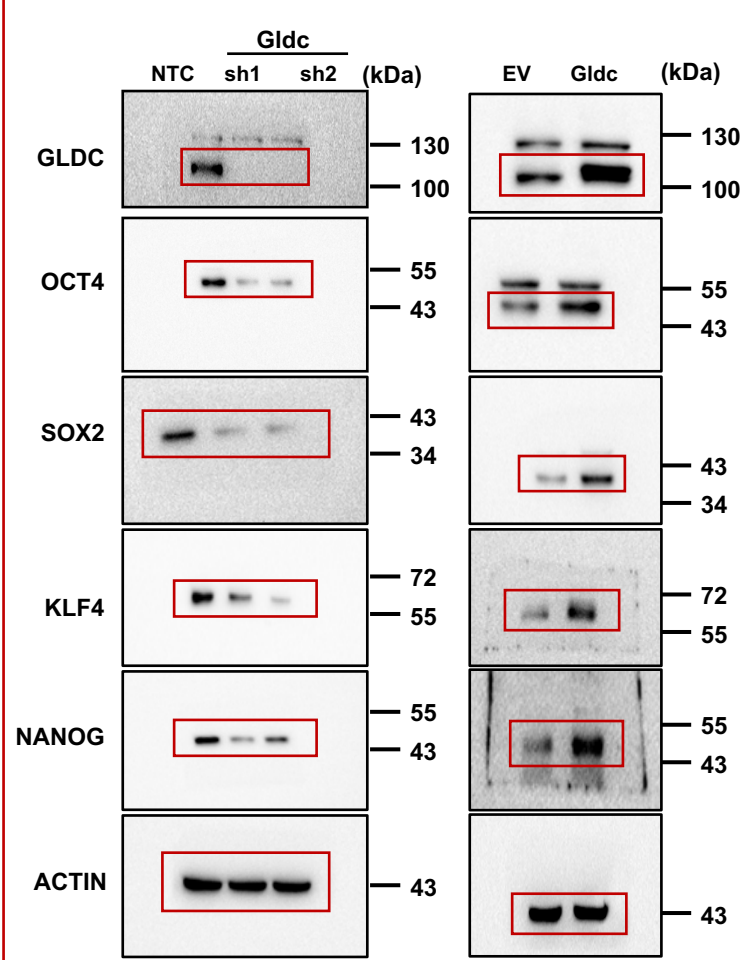

Fig 4E

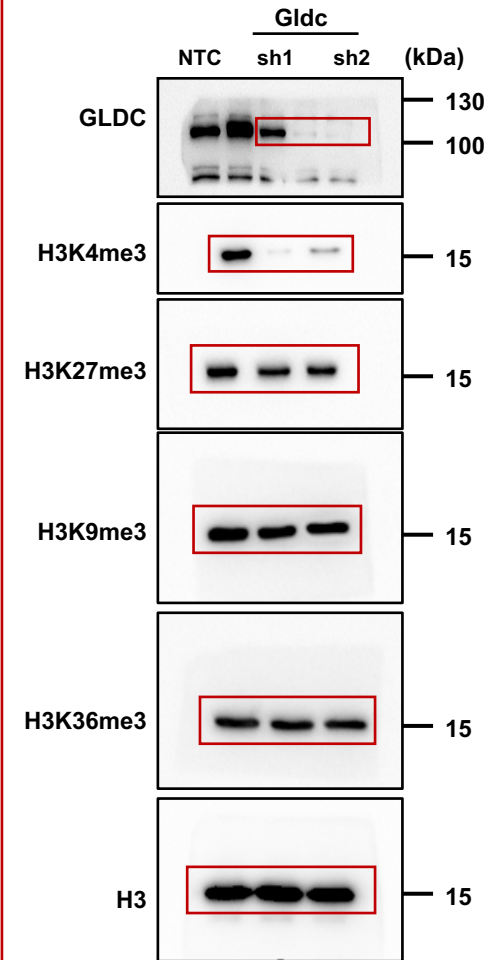

Fig 4G

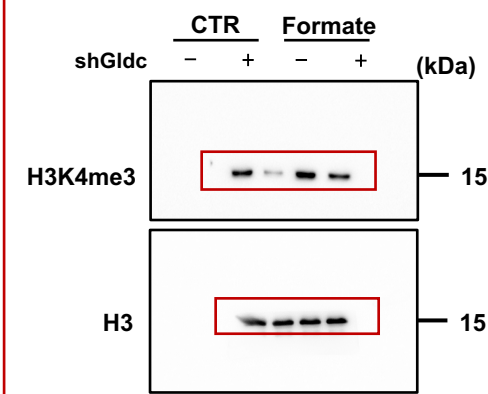

Supplement: Supplementary file 6 [file LSA-2019-00413_SdataF4.pdf]

Fig S4B

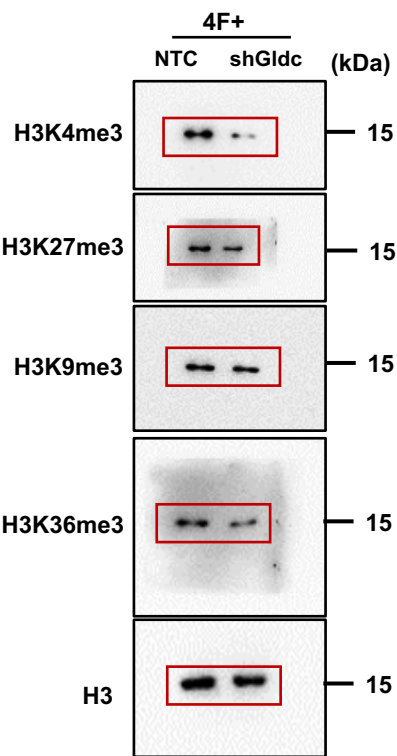

Fig S4D

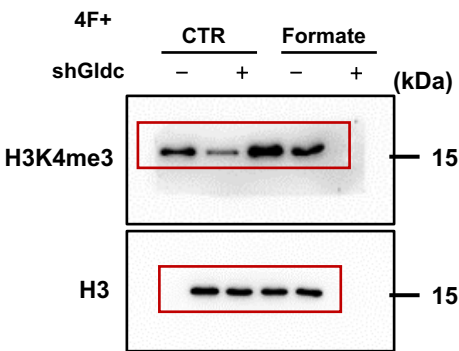

Supplement: Supplementary file 7 [file LSA-2019-00413_SdataFS4.pdf]

**Fig 5A**

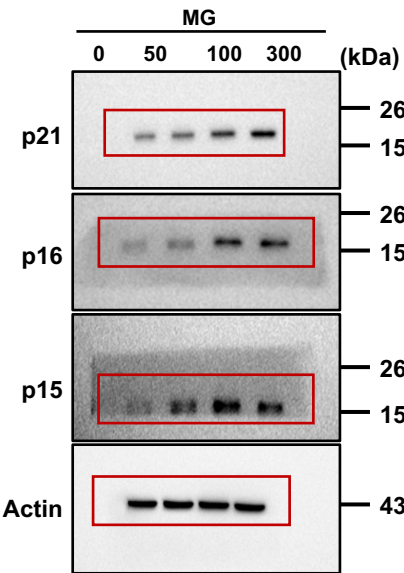

**Fig 5B**

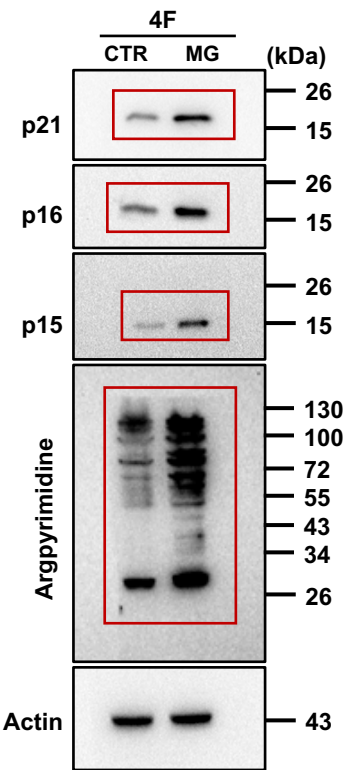

**Fig 5E**

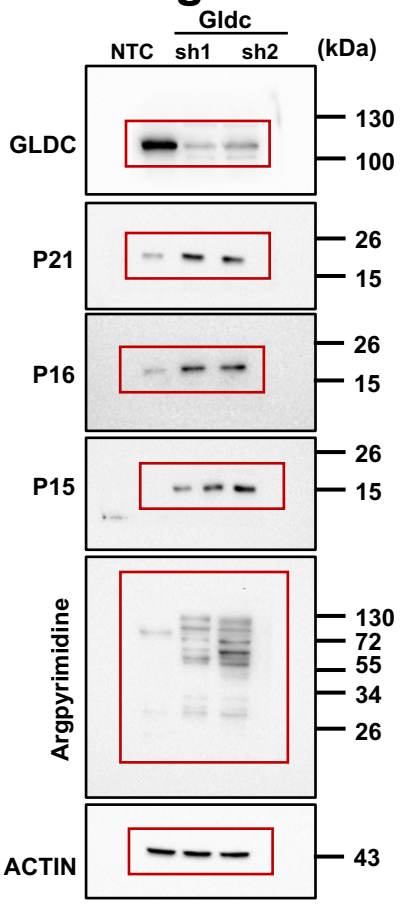

**Fig 5F**

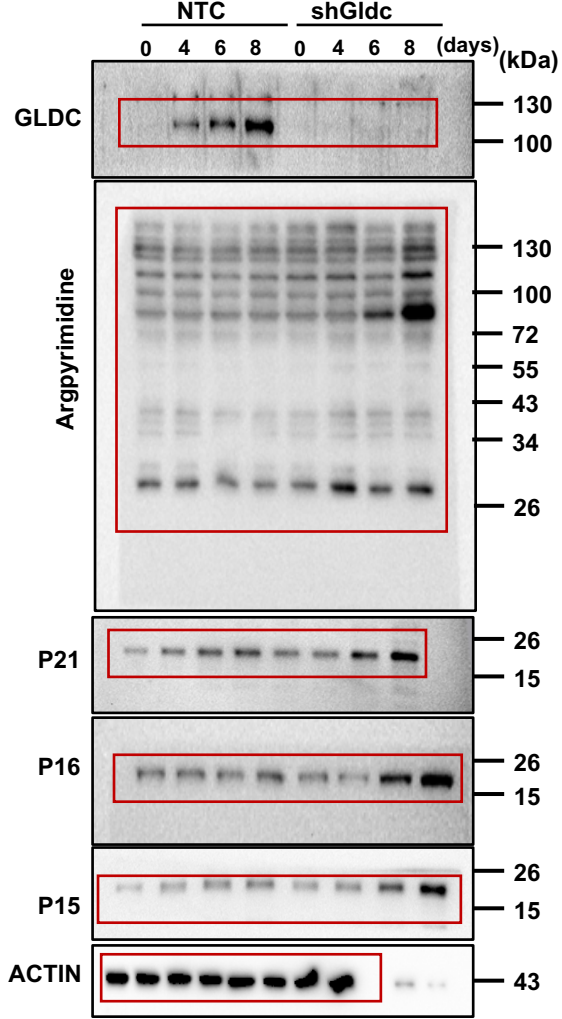

**Fig 5I**

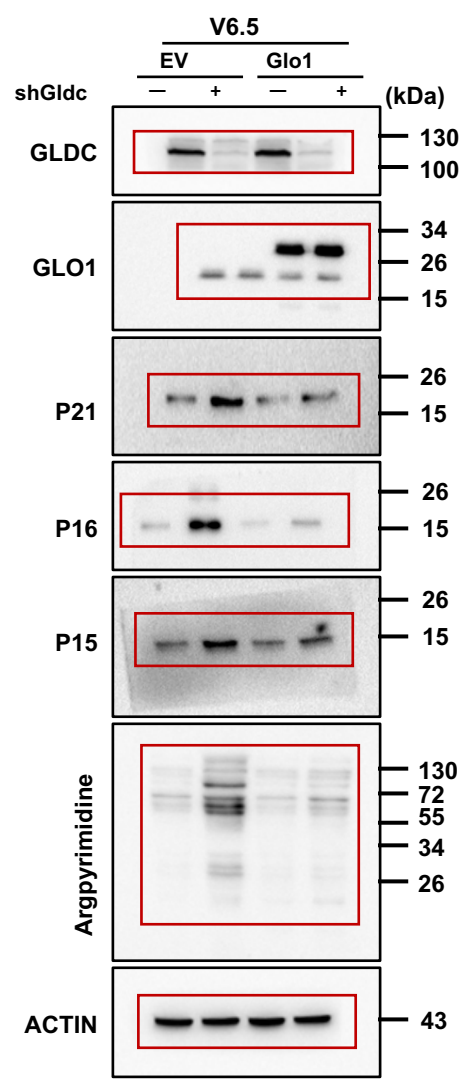

**Fig 5J**

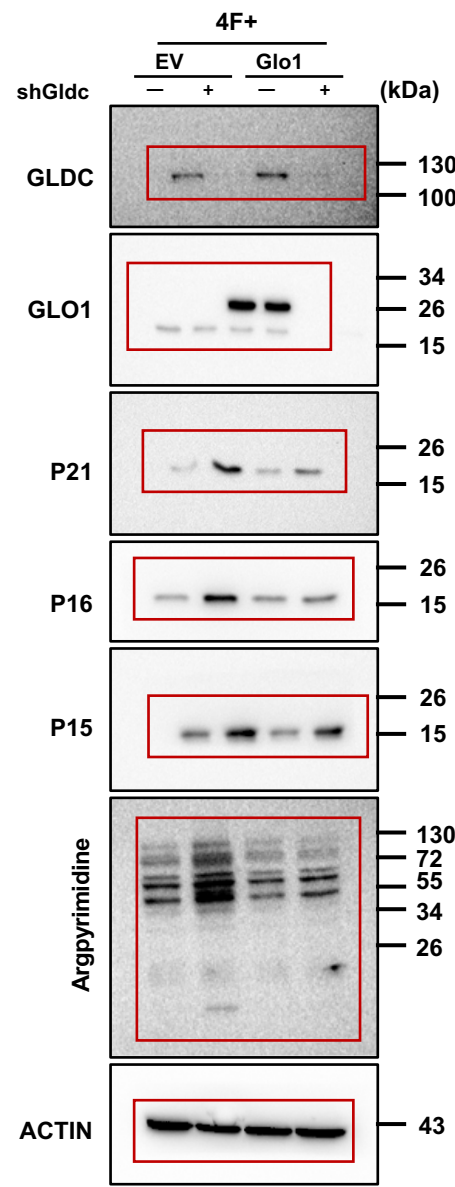

Supplement: Supplementary file 8 [file LSA-2019-00413_SdataF5.pdf]

**Fig S5A**

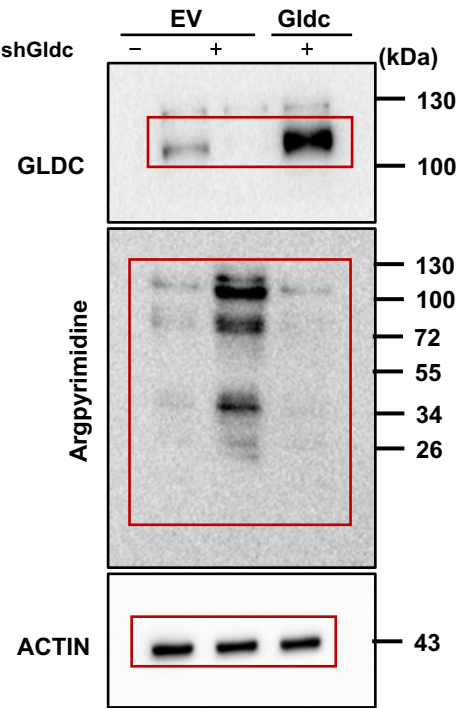

Supplement: Supplementary file 9 [file LSA-2019-00413_SdataFS5.pdf]

**Fig 6B**

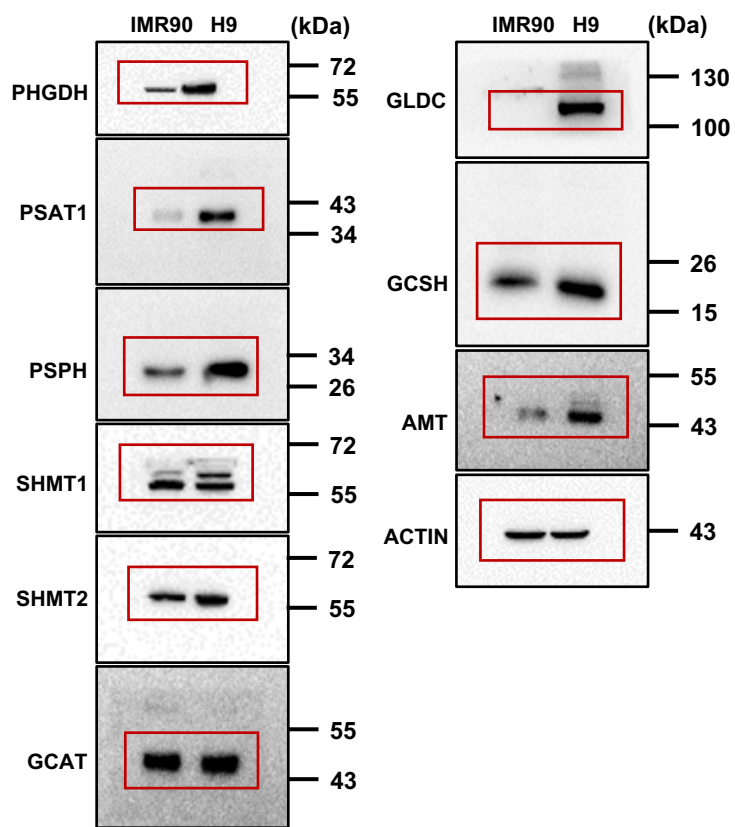

**Fig 6D**

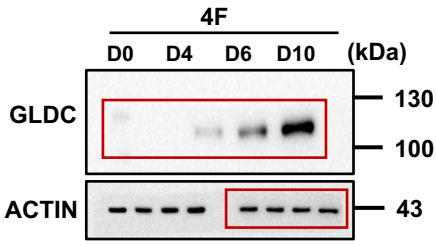

**Fig 6E**

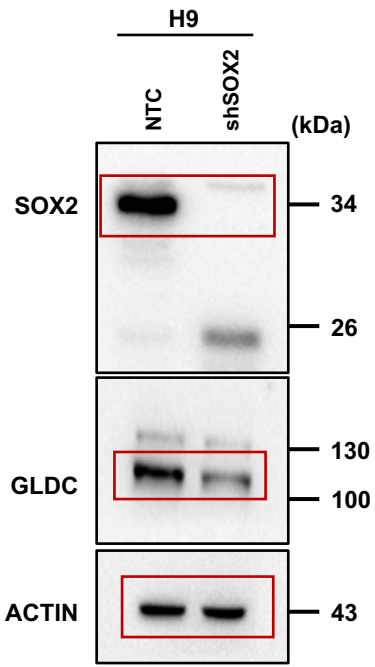

**Fig 6F**

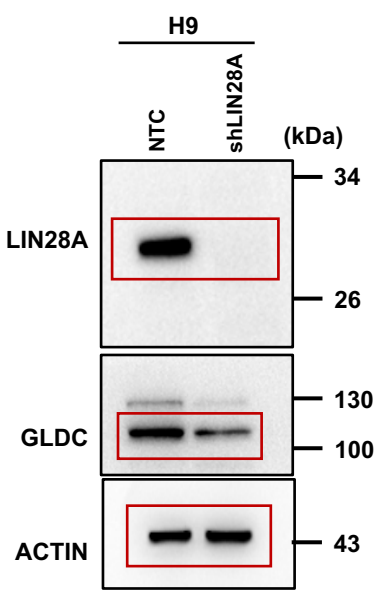

**Fig 6G**

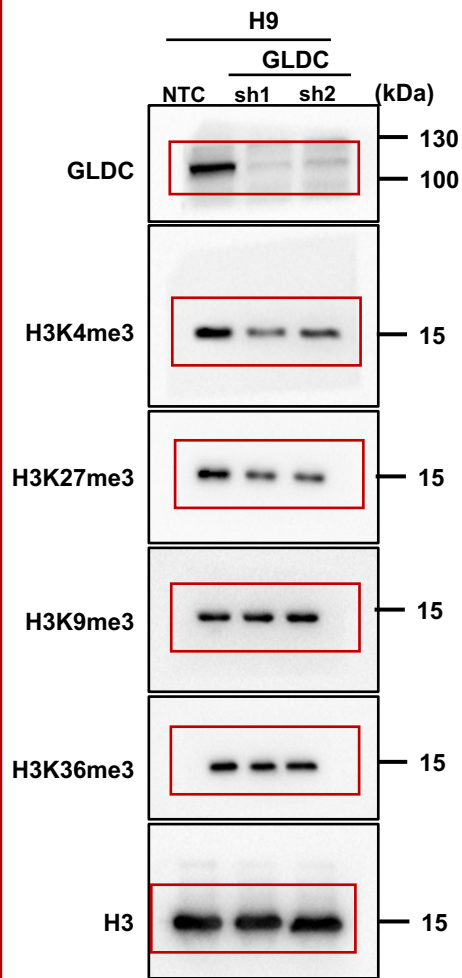

**Fig 6H**

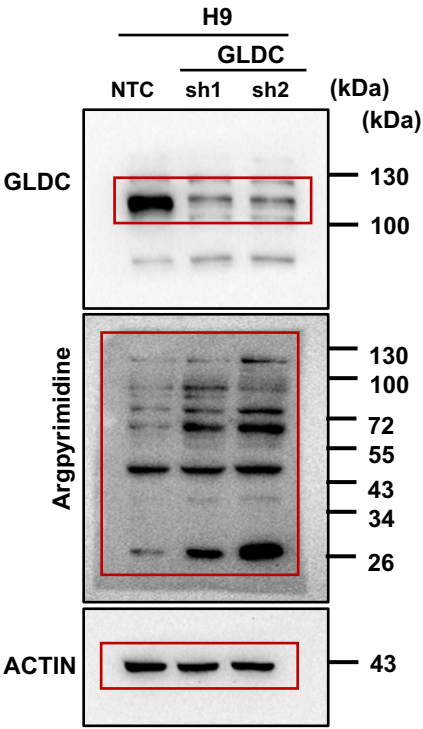

**Fig 6I**

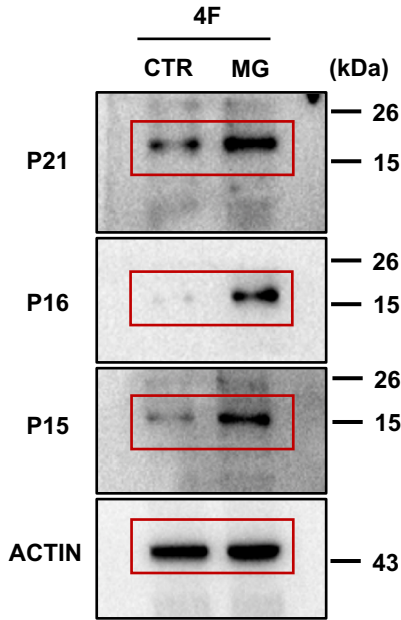

**Fig 6J**

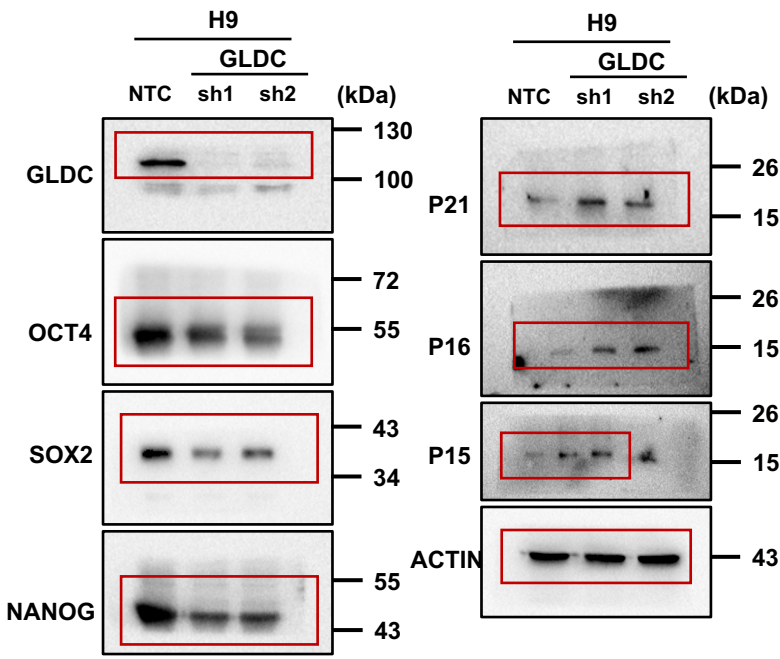

Supplement: Supplementary file 10 [file LSA-2019-00413_SdataF6.pdf]
